# Experimental Study on Shear Behavior of Rock Composite Material under Normal Unloading Conditions

**DOI:** 10.3390/ma16031233

**Published:** 2023-01-31

**Authors:** Bo Liu, Yifan Chen, Hang Lin, Rihong Cao, Shengwen Zhang

**Affiliations:** School of Resources and Safety Engineering, Central South University, Changsha 410083, China

**Keywords:** normal unloading, joint, roughness, DIC, acoustic emission, energy

## Abstract

As a composite material, the stability of rock mass is usually controlled by a joint. During the process of excavation, the normal stress of the joint decreases continuously, and then the shear strength of the joint decreases, which may eventually lead to the instability and failure of rock mass. Previous studies have mainly focused on the shear behavior of joints under constant normal stress, but have rarely considered the unloading of normal stress. In this paper, a direct shear test of joints with different roughness was carried out, in which the shear stress remained unchanged while the normal stress decreased. The strength characteristics of joints were explored, and the deformation and acoustic emission-counting characteristics of joints were analyzed by digital image correlation (DIC) techniques and acoustic emission (AE). A new method for predicting the instability of joints under normal unloading was proposed based on the evolution law of normal deformation energy (U_n_), tangential deformation energy (U_s_) and total deformation energy (U_0_). The results show the following: (1) The unloading amount of normal stress was enlarged for greater initial normal stress and roughness, while it decreased with an increase in initial shear stress. (2) AE events reached their maximum when the normal stress was equal to the failure normal stress, and the *b*-value fluctuated more frequently in stable development periods under normal unloading conditions. (3) U_0_ would change with the loading and unloading of stress, and this may be used to predict the unloading instability of rock mass using the abrupt change of U_0_.

## 1. Introduction

As the weak plane in rock mass, the joint is usually the key factor in the stability of instances of rock mass engineering such as slopes, dam foundations and underground chambers [1,2,3], thus further affecting human life and property as well as the environment [4,5,6]. Research on the deformation and strength characteristics of joints aims to provide a basis for the evaluation and utilization of rock mass stability [7,8]. In view of this, many scholars have systematically carried out a large number of studies on the shear strength and deformation characteristics of joints from theoretical derivations [9,10], laboratory tests [11,12,13,14,15] and numerical simulations [16,17,18]. The research content includes the effect of size, roughness, freeze–thaw action, infilled material, bolt mode, etc. on shear strength of joints. It can be seen that these studies are all based on constant normal stress or stiffness, but rarely considered the engineering problems caused by the unloading of normal stress. However, due to the discontinuity and heterogeneity of rock, the mechanical properties of rock vary greatly under loading and unloading conditions [19]. Therefore, various uniaxial loading and unloading tests or triaxial unloading tests have been carried out, achieving fruitful results. Duan, Ji [20] used granite to carry out laboratory tests, and radial unloading tests showed that radial strain played a major role in the change of bulk strain during unloading. Wang, Li [21] explored the energy storage and conversion characteristics of sandstone in the process of discontinuous loading and unloading, and explained the strength law of rock from the perspective of energy. Rong, Li [22] found that different aspect ratios would affect the failure mechanism of rock mass during unloading, and that an increase in aspect ratio would change the failure mode of rock from splitting failure to tensed shear failure. Xiao, Yu [23] studied the influence of temperature on the failure process of sandstone, and believed that Young’s modulus in the unloading stage first increased and then decreased with an increase in temperature. Undoubtedly, these studies have made people more deeply understand the deformation, strength characteristics, fracture evolution mechanism and energy evolution law of rock mass under unloading conditions [24,25,26,27].

Nevertheless, the above studies are limited to the unloading failure of rock mass under compression conditions only. In fact, during the excavation of deep rock mass, the shear strength of the potential fracture surface will decrease due to the decrease in normal stress [28,29]. When the slope breaks along the sliding plane, the normal stress also decreases in the direction perpendicular to the sliding plane. Therefore, it is necessary to study the shear mechanical behavior of rock mass under normal unloading condition. Regrettably, there is little coverage of this. Huang, Guo [30] carried out an experimental study on the failure characteristics of sandstone with a single fracture. Based on Huang, Guo [30], Zhao et al. carried out unloading shear tests on rock mass with multiple fractures, and found that the relative roughness coefficient of the fracture surface was larger when the rock mass failed under normal unloading conditions [31]. Zhu and Huang [32] performed unloading shear tests of complete rocks. It can be seen that these studies mostly focus on intact rock or non-penetrating fissure rock mass without considering the important influence of joints on rock stability, especially the deformation and strength characteristics of joints under normal unloading conditions. Their research methods are also relatively simple. Usually, AE and DIC technology are combined to study the damage evolution process of rock mass [33,34,35]. By analyzing AE parameters and the whole process of rock mass surface deformation provided by DIC technology, the microscopic mechanism of rock mass under normal unloading conditions can be better understood. In addition, due to the isolation of rock samples, their stress–strain state is complex, so it is difficult to take the stress–strain state as the only instability criterion [36,37]. Therefore, analysis of energy and stress–strain state should be combined to better reveal the failure characteristics of rock mass [38,39]. 

On this basis, this paper intends to use digital image correlation (DIC) techniques and an acoustic emission (AE) system to carry out a shear test of rock mass under normal unloading conditions. The deformation characteristics, strength, acoustic emission characteristics and energy evolution law of rock mass during the unloading process were analyzed, and the effects of initial normal stress, initial shear stress and the different roughness of joints on the shear mechanical behavior of rock mass were investigated. The research results further enrich the mechanics of unloading rock mass and provide some reference for the instability failure and disaster prevention of unloading rock mass under normal unloading conditions. 

## 2. Materials and Methods

### 2.1. Specimen Preparation

**Figure 1:**
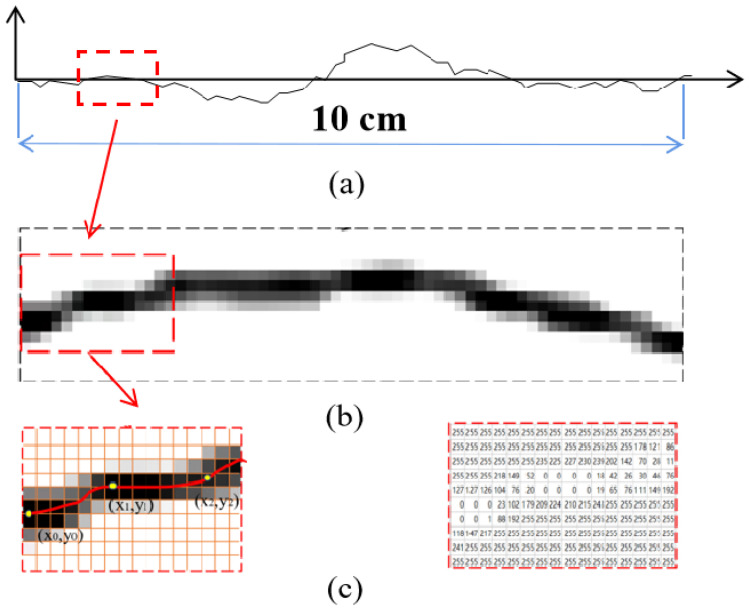
Extraction of coordinate points. (**a**) standard JRC profile with value of 18–20 (**b**) basic gray image (**c**) gray matrix and intensity matrix.

**Figure 2:**
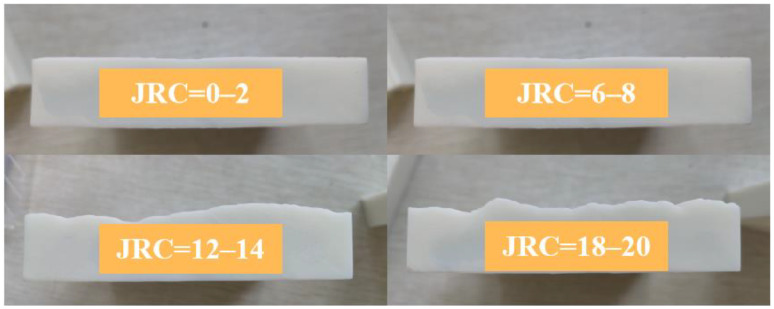
Different JRC molds.

Firstly, coordinate points of a Barton curve were extracted. In this paper, the gray image processing method was used to obtain the coordinates of the standard contour. In this way, the data accuracy was higher and the sampling spacing could be set flexibly. Taking the outline of JRC as 18–20 as an example, the operation process is explained in detail: (1) Import the picture containing the outline into Matlab to obtain the basic gray level image (Figure 1b); (2) The built-in function of Matlab then divided the grayscale image into the intensity matrix (Figure 1c). The width of the grid was 16 pixels, the height was 10 pixels, equalling a total of 160 pixels. Each pixel was represented by a grayscale value ranging from 0 to 255, and the darker the color in the grid, the smaller the corresponding intensity value; (3) Select the grid in which the minimum value of each column was located and calculate the corresponding coordinate points of the grid; (4) Calculate the coordinate points of each grid column successively to obtain the coordinates of the contour.

Assuming that the gray matrix corresponding to the image is of dimensions *m* × *n*, and the total length of the standard contour is 10 cm, then the width p of each column of the gray matrix is:

(1) $$p=\frac{10}{m-1}$$

Since the row spacing and column spacing between each two grids in the grayscale matrix are the same, the total height *q* of the grayscale matrix is:

(2) $$q=\frac{10}{m-1}\times (n-1)$$

Then, the corresponding coordinates of the *i*-th row and the *j*-th column in the grayscale matrix are:

(3) $$\begin{array}{l} x_{ij}=(i-1)\times \frac{10}{m-1}\\ y_{ij}=(j-1)\times \frac{10}{m-1} \end{array}\}$$

where, *x_ij_* and *y_ij_*, respectively represent the abscissa and ordinate of row *i* and column *j*.

According to the above principle, the coordinate of each gray matrix corresponding to the grid is obtained.

The corresponding roughness two-dimensional curve can be obtained by importing the coordinate points obtained by Matlab into CAD, and the corresponding roughness of the joint can be obtained by importing the obtained two-dimensional curve into 3DMAX. The image file was handed over to the manufacturer, and the molds with different roughness were made in batches. Resin joints with JRC equal to 0–2, 6–8, 12–14, 18–20 are shown in Figure 2, respectively. The thickness of the mold is 2 cm.

**Figure 3:**
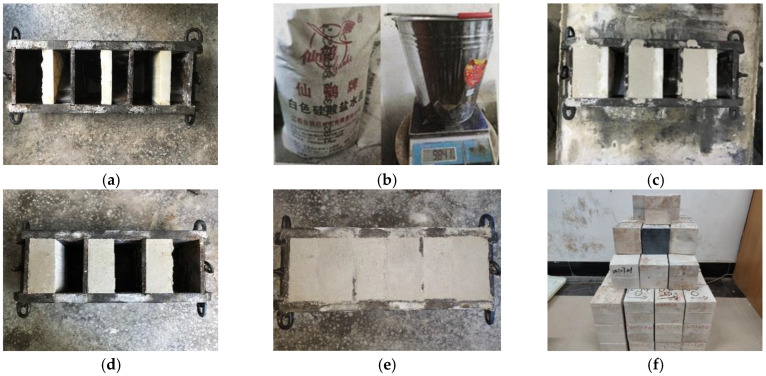
The production process of the specimen. (**a**) Installation of mold. (**b**) Weighing of raw material. (**c**) Casting and vibration. (**d**) Pouring of the bottom half of the specimen. (**e**) Complete specimen. (**f**) Cured specimen.

In this paper, the shear mechanical behavior of rock mass with different roughness joints under normal unloading conditions was investigated. However, the shape of natural joints was complex and changeable; this meant that they were neither repeatable nor conducive to the summary and induction of test rules [32,40]. Therefore, cement mortar and river sand were selected as test materials [41]. The specific production steps are as follows: (1) The assembly of the mold. The impurities of the steel plate die and resin joint were cleaned first, and then the resin joint was embedded into the steel plate die (Figure 3a). (2) The making of cement mortar. The raw materials were weighed according to the mass ratio of cement, sand and water equal to 2:1:0.75, and then all the raw materials were poured into the container for stirring. The stirring was stopped when no large particles could be seen on the surface of the slurry and the slurry showed a fluid-plastic shape (Figure 3b). (3) The casting of the specimens. Firstly, the assembled mold was coated with appropriate oil, and then the mixed cement mortar was injected into the mold in layers. After pouring, the mold was placed on the shaking table and the specimen was vibrated at a certain rate. After 3 h, the excess cement mortar on the surface of the initial setting specimen was removed with a shovel. After 24 h, the solidified specimen was demolded, and the specimen was placed in the mold for the casting of the specimen footwall (Figure 3c,d). (4) The curing of the specimen. The prepared rock mass with different roughness joints was placed in indoor maintenance for 28 days, waiting for the follow-up experimental study (Figure 3f). The specific production process is shown in Figure 3. The size of the specimen is 10 cm × 10 cm × 10 cm.

To facilitate the determination of subsequent test schemes, a uniaxial compression test of standard specimens and a direct shear test of joints with different roughness were carried out. The uniaxial compressive strength of the three standard cylindrical specimens was 32.2, 30.2, 28.5 MPa, and the average value of the three specimens (30.3 MPa) was taken as the uniaxial compressive strength of the specimens. The cohesion and internal friction angle of joints with different roughness are shown in Table 1.

### 2.2. Loading Mode

**Figure 4:**
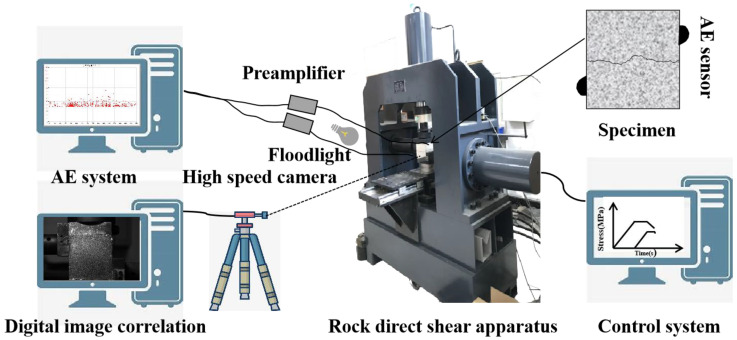
Test instrument.

The test system includes YZW100 multi-functional rock direct shear apparatus, an AE system and a DIC system, as shown in Figure 4. The direct shear tester adopts bidirectional pressure servo control; the maximum compression load of both vertical and horizontal axes is 500 kN, which can accomplish stress control and displacement control in two ways. The AE system sets the threshold to 40 dB. The DIC system consists of a floodlight, a high-speed camera and an image acquisition computer. The high speed camera can obtain the speckle image of the object in each deformation stage in real time, and then use DIC technology to calculate the mechanical parameters of the object surface deformation point. During the experiment, eight images were taken every second. The digital image of the whole loading process obtained by the high speed camera is processed by a computer analysis system to elucidate the evolution process of the strain field [34,42].

**Figure 5:**
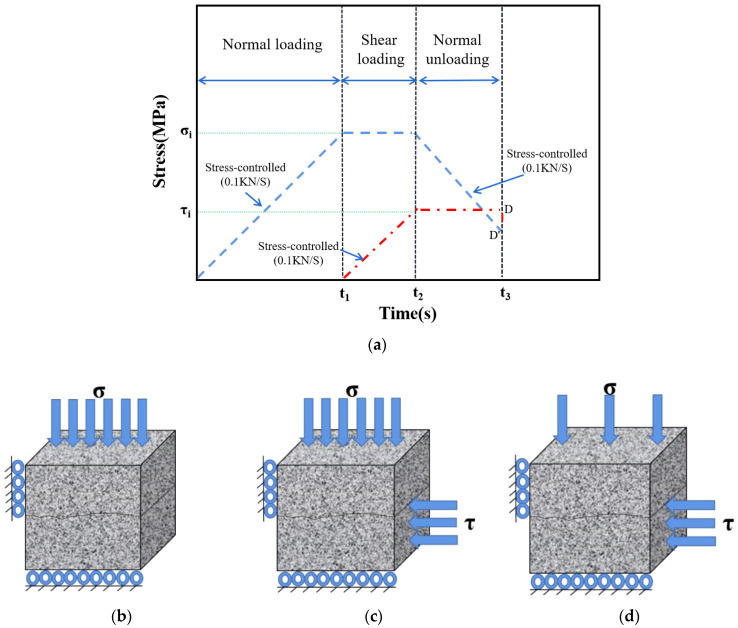
Stress path in the experimental process. (**a**) Loading process. (**b**) Step 1. (**c**) Step 2. (**d**) Step 3.

Stress control was adopted in the loading and unloading process of this test, and the loading and unloading rates were all 0.1 kN /s. The test stress path was divided into three steps [32], as shown in Figure 5.

Step 1: normal force Fn is applied until initial normal stress σ_i_ at a loading rate of 0.1 kN/s. To ensure that no damage occurs during the application of normal force, σ_i_ should be less than the uniaxial compressive strength [43].

Step 2: tangential force Fs is applied until initial shear stress τ_i_ at a loading rate of 0.1 KN/s. The normal force should be kept constant during this process. To ensure the specimen’s failure during unloading, τ_i_ should be slightly less than the shear strength corresponding to the minimum initial normal stress σ_imin_ in the test scheme.

Step 3: the initial shear stress τ_i_ is kept constant, and the normal stress is unloaded slowly at a unloading rate of 0.1 kN/s until the specimen fails. Due to the stress control method adopted in the test, when the specimen fails, the curve drops sharply (D and D′ in Figure 5a).

To further explore the influence of initial normal stress, initial shear stress and the shear mechanical behavior of joints with different roughness, the test was divided into two groups, as shown in Table 2.

To reduce the test error, two specimens were made under each stress state.

## 3. Results and Discussion

### 3.1. Deformation Characteristics

In order to further understand the principal strain characteristics of the rock mass surface under normal unloading conditions, to study the mechanical characteristics of rock mass during unloading shear, a DIC system was used to analyze the principal strain variation rule and distance characteristics of the rock mass surface under different test conditions in this section. It should be noted that DIC was recorded when the shear stress was loaded.

**Figure 6:**
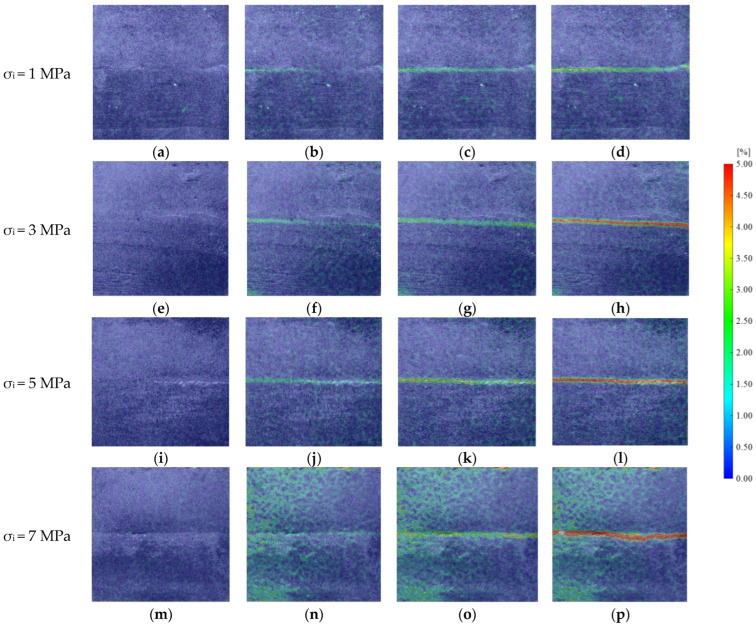
Principal strain field of specimens at different initial normal stress. (**a**) Frame 0. (**b**) Frame 480. (**c**) Frame 963. (**d**) Frame 1442. (**e**) Frame 0. (**f**) Frame 725. (**g**) Frame 1398. (**h**) Frame 2736. (**i**) Frame 0. (**j**) Frame 1046. (**k**) Frame 3329. (**l**) Frame 4792. (**m**) Frame 0. (**n**) Frame 1652. (**o**) Frame 3840. (**p**) Frame 6139.

In the group A, the initial shear stress was 0.7 MPa. In order to observe the experimental phenomenon more clearly, samples with JRC 0–2 were selected to analyze the variation rule of the rock mass principal strain field under different initial normal stress conditions. As shown in Figure 6, when the rock mass was not subjected to shear stress, the principal strain distribution was uniform and basically 0. With an increase in shear stress, the principal strain on the rock mass surface started to become uneven; the principal strain on the left side of rock mass first increased by about 1.25%. With the passage of time, the uneven zone gradually extended to the right, and the color of the uneven zone on the left became darker, which meant that the principal strain on the left side kept increasing. The normal stress continued to unload, and finally, the rock mass became unstable. At this time, a high strain zone with a principal strain of up to 5% appeared along the joint. Due to the different initial normal stress of rock mass, the principal strain law of rock mass was different in the process of shear loading and normal unloading. With an increase in the initial normal stress, the time for the uneven strain distribution on the surface of the rock mass became longer, and the high strain band when the rock mass was finally destabilized became wider, which indicated that the normal stress inhibited the deformation of the rock mass in the shear direction. However, when the final instability occurred, the energy was released rapidly and transferred to the adjacent rock mass along the joint.

**Figure 7:**
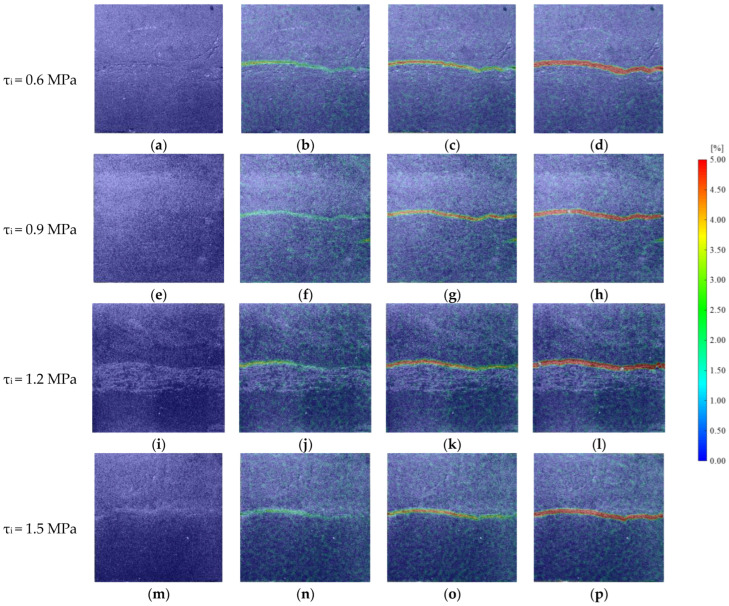
Principal strain field of specimens at different initial shear stress. (**a**) Frame 0. (**b**) Frame 1458. (**c**) Frame 2793. (**d**) Frame 3481. (**e**) Frame 0. (**f**) Frame 1279. (**g**) Frame 2341. (**h**) Frame 3152. (**i**) Frame 0. (**j**) Frame 1120. (**k**) Frame 2016. (**l**) Frame 2947. (**m**) Frame 0. (**n**) Frame 942. (**o**) Frame 1846. (**p**) Frame 2740.

In the group B, the maximum initial shear stress was up to 1.5 MPa. Therefore, samples with JRC of 12–14 were selected to analyze the variation law of the principal strain field under different initial shear stress conditions. Before the shear stress was applied, the color distribution on the surface of the rock mass was uniform in Figure 7. With the loading of the shear stress, an uneven strain band began to appear on the left and gradually expanded to the middle, and the value of the principal strain decreased from left to right. After the shear stress reached the initial value, the normal stress began to be unloaded, and the uneven strain zone continued to expand up to the surface of the connected rock mass. At this time, the rock mass was still not unstable, but the value of the main strain zone kept expanding, which was basically 3.5–4.5%. As the normal stress continued to unload, the high strain zone penetrated the surface of the rock mass and the rock mass became unstable. With an increase in initial shear stress, the non-uniform zone appeared earlier.

**Figure 8:**
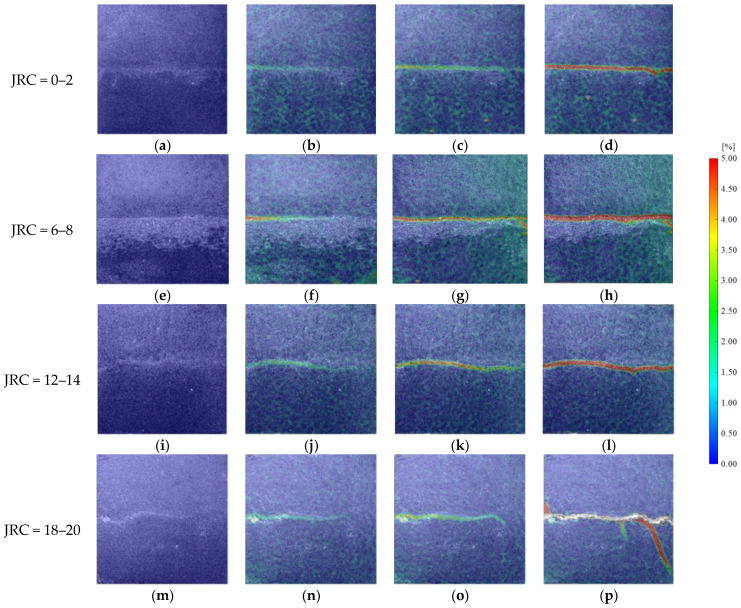
Principal strain field of specimens at different JRC. (**a**) Frame 0. (**b**) Frame 389. (**c**) Frame 672. (**d**) Frame 943. (**e**) Frame 0. (**f**) Frame 682. (**g**) Frame 1267. (**h**) Frame 1962. (**i**) Frame 0. (**j**) Frame 942. (**k**) Frame 1846. (**l**) Frame 2740. (**m**) Frame 0. (**n**) Frame 1253. (**o**) Frame 2158. (**p**) Frame 3072.

Figure 8 shows the principal strain diagram of rock mass with different roughness with an initial normal stress of 3 MPa and an initial shear stress of 1.5 MPa. As can be seen from Figure 8, when the shear stress was not loaded, the surface strain of the rock mass was evenly distributed. After a period of time after the shear stress was loaded, the strain near the joint began to change and the color distribution on the nephogram began to be uneven. The strain on the left side of the joint increased first, and then the main strain on the right side increased. The principal strain near the whole joint was about 1.5%. With the continuous unloading of normal stress, a high strain zone appeared near the joint, and the high strain value in this region was about 3.0~4.5%; meanwhile, the strain in the remaining region was basically maintained at the initial 0.5%. The last picture was taken when the rock mass was unstable. It can be seen that the rock mass eventually failed along the high strain zone; namely, through-through failure occurred along the joint. With different JRC, there was little difference in the final formation of high strain bands—which basically extended along the joint—but the final penetrating time would be extended. In addition, the high strain band extended downward when JRC was equal to 18–20.

**Figure 9:**
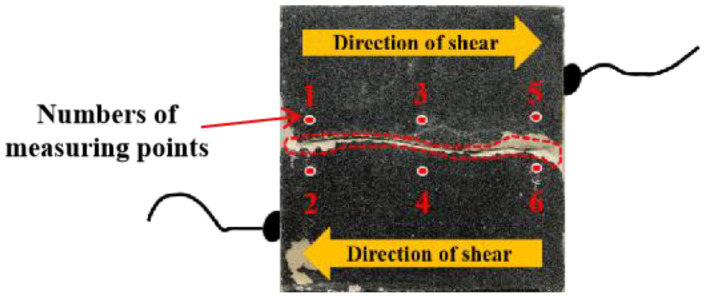
Location map of measuring points.

According to Figure 9, six points were constructed to obtain displacement information on both sides of the joint during the whole process, so as to fully understand the failure process of the joint [44]. The distance between each group of measuring points is about 2 cm, and the measuring points are symmetrically distributed on both sides of the joint. This setting allows the measuring point to be as close to the joint as possible while being higher than the convex point of the joint. When setting up the measuring point, the connection of the measuring point should be perpendicular to the joint as far as possible. In the software, the relative displacement of each pair of measuring points in the X and Y directions in the whole process of the test was firstly derived, and then the relative displacement change in the X and Y directions was transformed into the relative displacement change along the line direction (normal) and vertical line direction (tangential) by using the relative position relationship of each pair of monitoring points. The smaller the relative displacement of the X and Y directions was, the more likely the joint was to be destroyed at the bottom of the bump, that is, the stronger the cutting effect is; otherwise, the stronger the climbing effect is. In the figure, 1-2-X refers to the change of relative distance between measuring points No. 1 and No. 2 in the X direction during the test (the Y direction was expressed in the same way). After the joint was damaged, the worn part of joint can be clearly observed, as shown in Figure 9.

**Figure 10:**
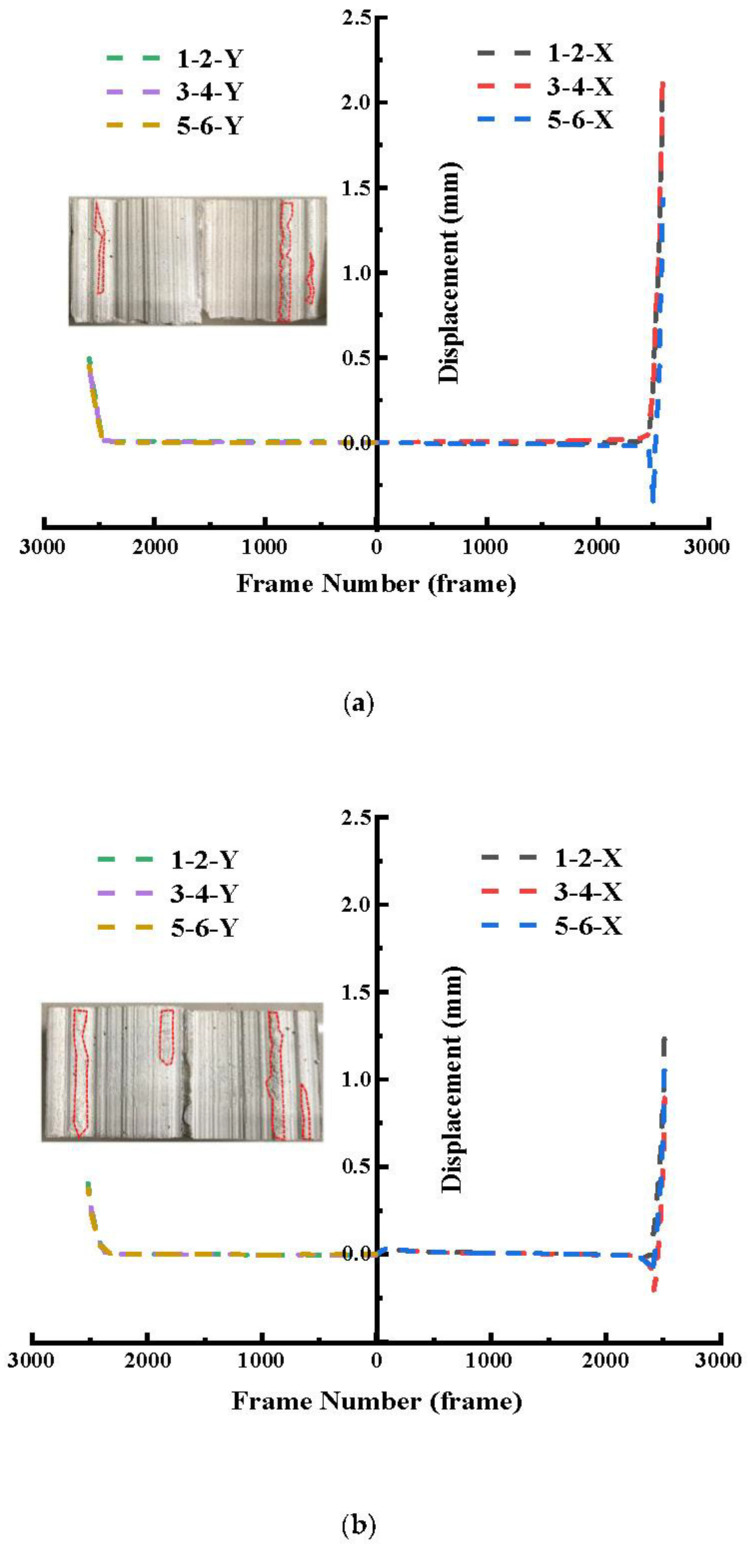

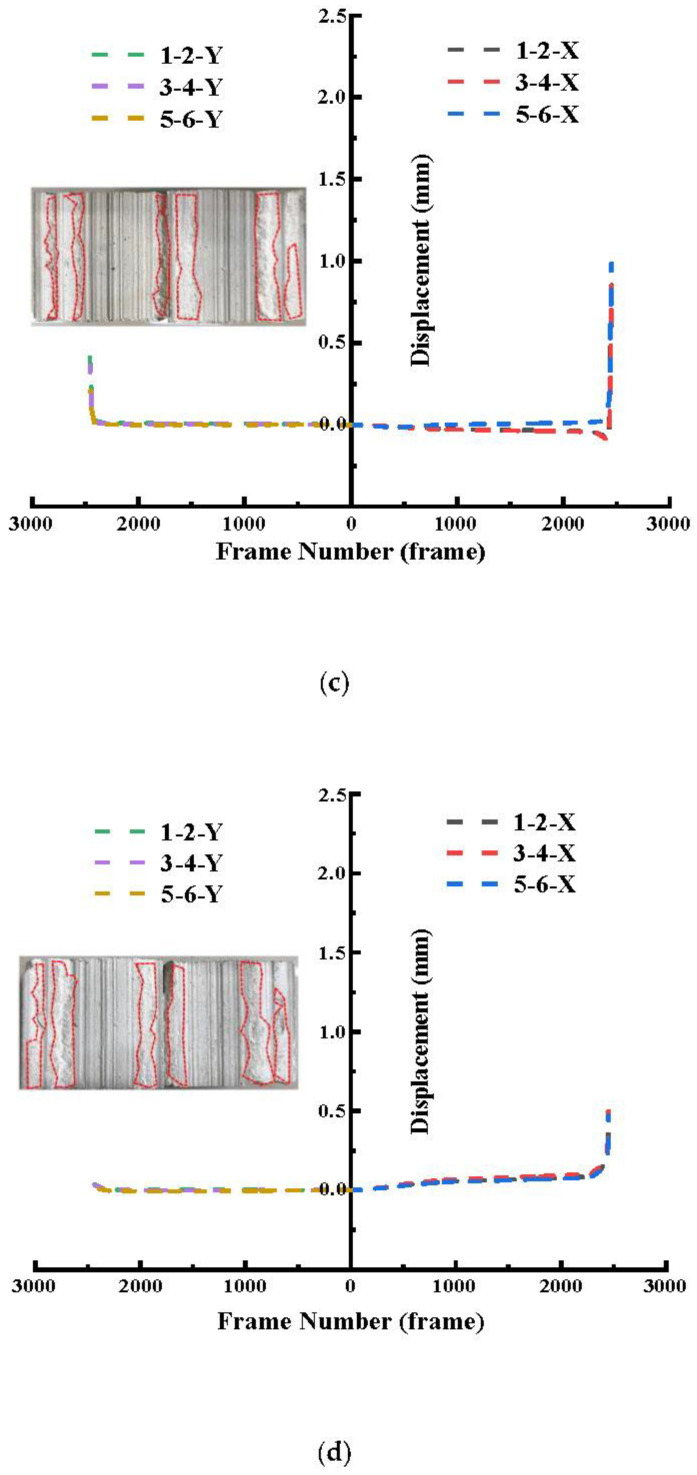
Relative displacement changes of measuring points in X and Y directions under different initial shear stress conditions. (**a**) τ_i_ = 0.6 MPa. (**b**) τ_i_ = 0.9 MPa. (**c**) τ_i_ = 1.2 MPa. (**d**) τ_i_ = 1.5 MPa.

It can be seen from Figure 10 that before the failure of the joint, the displacement in the X direction between the measured points did not change significantly. As force-controlled stress loading and unloading was adopted in this test, the joint was destroyed suddenly. When the joint was damaged, the relative displacements in the X direction between the measuring points rose sharply, and the relative displacements of the three pairs of measuring points were basically the same. The average value of the relative displacement of three pairs of measuring points in the X direction was taken to measure the displacement change of measuring points in the X direction, which was negatively correlated with τ_i_ when the joint finally failed. When τ_i_ was 0.6 MPa, the average relative displacement in the X direction of the three pairs of measuring points was 1.92 mm, and when τ_i_ increased to 0.9 MPa, 1.2 MPa and 1.5 MPa, the average relative displacement in the X direction of the three pairs of measuring points decreased to 1.15 mm, 0.89 mm and 0.48 mm, respectively. Similarly, the relative displacement of each pair of measuring points in the Y direction was analyzed under different initial shear stress conditions, and was similar to the change rule of relative displacement in the X direction. Before the failure of the joint, the relative displacement in the Y direction was very small under different initial shear stress conditions, and the displacement basically did not change with the increase in time. With the continuous unloading of normal stress, the joint suddenly broke down, and the relative displacement of each measuring point in the Y direction increased suddenly. The average relative displacement of each measuring point in the Y direction under each test condition was taken as the displacement of the specimen in the Y direction under this condition. When τ_i_ was 0.6 MPa, the average relative displacement in the Y direction was 0.47 mm. With the increase in the initial shear stress, the average relative displacement in the Y direction decreased to 0.38, 0.32 and 0.1 mm. That is, the increase in τ_i_ will make the climbing effect weaker and the cutting effect stronger. The final wear area of the joint also increased with the increase in τ_i_ (the larger the area marked red in Figure 10).

**Figure 11:**
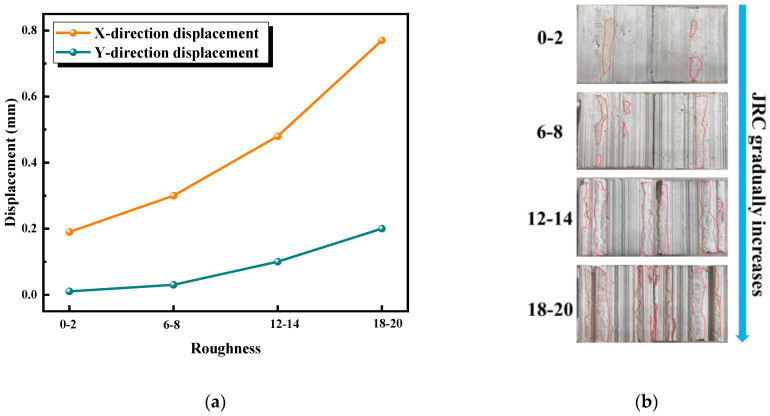

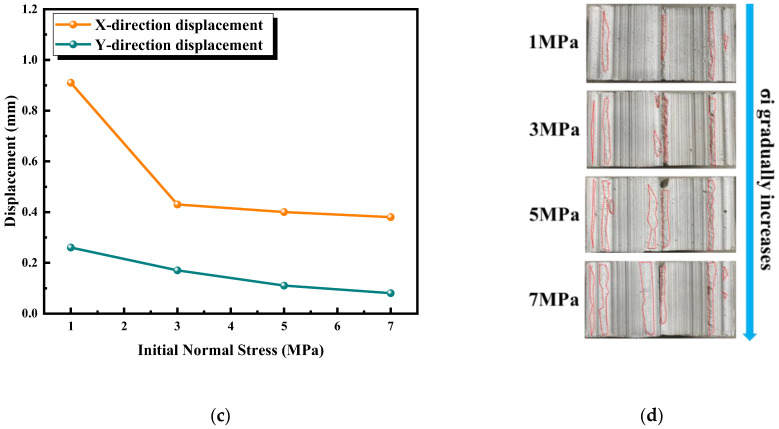
Relative displacements in the X and Y directions of different initial normal stress and roughness. (**a**) Relative displacements in the X and Y directions under different roughness conditions. (**b**) Wear condition of the joint. (**c**) Relative displacements in the X and Y directions of different initial normal stresses. (**d**) Wear condition of the joint.

Figure 11a showed the average relative displacements of measuring points in the X and Y directions as a function of the roughness of the joint (taking σ_i_ = 3 MPa, τ_i_ = 1.5 MPa for example). When the JRC of the joint increased from 0–2 to 18–20, the relative displacement of each measuring point to the X and Y direction also increased gradually. When the roughness of joints was 0–2, the relative displacements of measuring points in the X and Y directions were only 0.19 and 0.01 mm; when the roughness of joints was 18–20, the relative displacements of measuring points in the X and Y directions increased to 0.77 and 0.20 mm. This phenomenon showed that the initial stress remained unchanged, and with the increase in roughness, the slope climbing effect and dilatancy phenomenon were more obvious in the shear process of the joint under normal unloading conditions, which was similar to the dilatancy characteristics of the normal stress shear test [45]. Figure 11b illustrates the relationship between the wear area of the joint and the roughness. It can be clearly seen from this figure that the rougher the joint was, the more prone the surface of the convex was to wear due to friction, and the wear area kept increasing. It should be noted that the convex on the joint was only slightly worn rather than being gnawed off in the whole shear process. Therefore, this did not mean that the more serious the wear was, the less the change in the relative distance between the measuring points would be when the joint was damaged. That is to say, this phenomenon is consistent with the results in Figure 11a. Figure 11c shows the changes in the average relative displacements in the X and Y directions with respect to the initial normal stress (τ_i_ = 1.5 MPa and JRC = 12–14) at the same initial shear stress and roughness of the joint. The relative displacements in the X and Y directions decreased with the increase in the initial normal stress. When the initial normal stress of the test was 1 MPa, the average relative displacements of the measured points along the X and Y directions were 0.91 mm and 0.26 mm. When the initial normal stress increased to 7 MPa, the average relative displacements of the measured points along the X and Y directions were only 0.38 and 0.08 mm. Combined with the relative displacements of measuring points along the X and Y directions, the climbing effect became weaker and the tooth cutting effect became stronger with the increase in the initial normal stress in the unloading shear process of the joint. On the other hand, with the increase in initial normal stress, the dilatancy effect of joints was inhibited. Figure 11d shows that the increase in initial normal stress would increase the wear area of joints [46].

### 3.2. Strength Characteristics

The Mohr–Coulomb shear strength equation is:

(4) τ=σtanφ+c

where σ is the normal stress (MPa) acting on the specimen, *φ* is the internal friction angle of the joint (°), c is the cohesion of the joint (MPa), and *τ* is the shear strength of the joint (MPa) under corresponding conditions.

It can be obtained from Equation (4):

(5) $$\sigma =\frac{\tau -c}{\tan\varphi }$$

**Figure 12:**
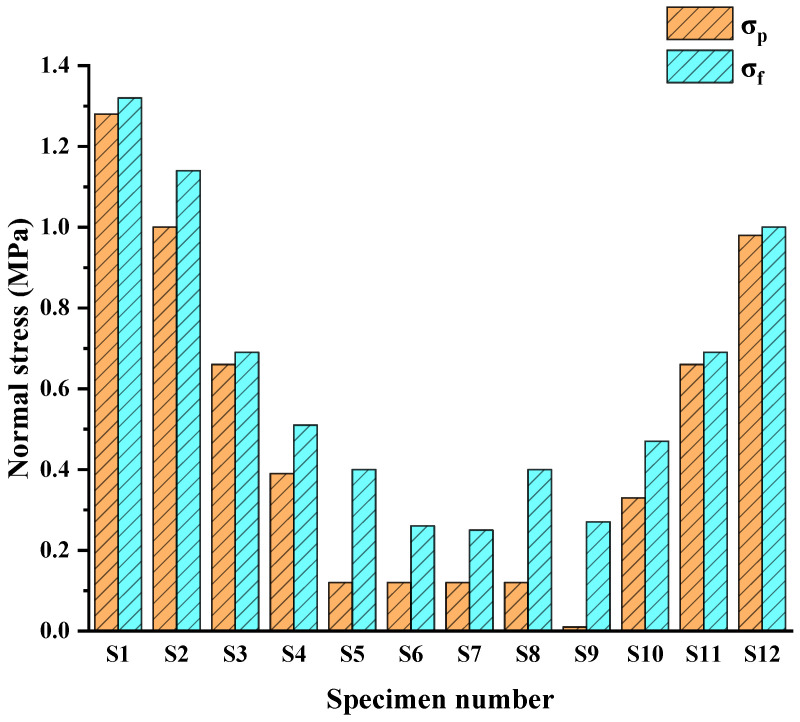
Theoretical normal stress σ_p_ and actual normal stress σ_f_ of unloading failure.

Let σ be equal to σ_p,_ and *τ* be equal to τ_i_; the c and *φ* of different roughness joints can therefore be seen in Table 1, where σ_p_ is the theoretical normal stress corresponding to the initial shear stress calculated according to the formula. As shown in Table 3, three groups of representative specimens with different initial normal stress, initial shear stress and roughness were, respectively, taken and numbered as S1–S12. According to Table 1, Table 3 and Equation (5), the theoretical normal stress σ_p_ of the selected specimen was calculated. The actual normal stress σ_f_ and the calculated theoretical normal stress σ_p_ are shown in Figure 12. 

Figure 12 shows that the theoretical normal stress of specimens S1–S12 was 1.28, 1.00, 0.66, 0.39, 0.12, 0.12, 0.12, 0.12, 0.01, 0.33, 0.66 and 0.98 MPa, respectively. However, the failure occurred when the normal stress was not unloaded to the calculated theoretical normal stress value. The actual normal stress of each joint was 1.32, 1.14, 0.69, 0.51, 0.4, 0.26, 0.25, 0.4, 0.27, 0.47, 0.69 and 1.00 MPa, respectively. Therefore, rock mass is more prone to failure under unloading conditions, and its strength will be weakened to a certain extent [47].

**Figure 13:**
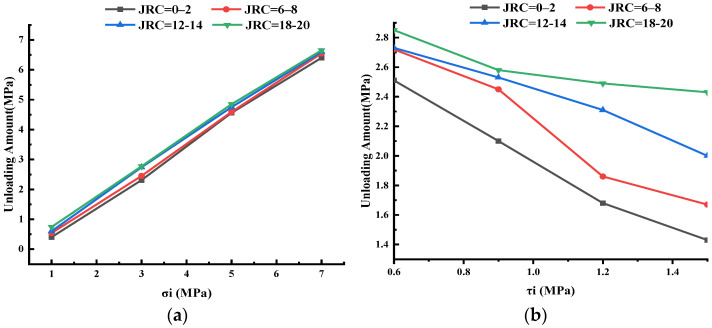
Unloading amount and initial stress. (**a**) Unloading amount and initial normal stress. (**b**) Unloading amount and initial shear stress.

Figure 13a illustrated the relationship between the unloading amount and the initial normal stress. With an increase in the initial normal stress, the unloading amount increased, and the relationship between them was basically linear. Taking the specimen with JRC equal to 6–8 as an example, when the initial normal stress was 1 MPa, the unloading amount was 0.54 MPa; meanwhile, when the initial normal stress increased to 7 MPa, the unloading amount was as high as 6.55 MPa, that was, the joint would be destroyed when the normal stress was unloaded to about 0.45 MPa. This is because when the roughness of the joint and the initial shear stress remained unchanged, the failure of the specimen was mainly controlled by the normal stress, that was, the specimen would basically fail after the normal stress was unloaded to a critical value. Figure 13b shows the initial shear stress–unloading curve, which generally showed a downward trend. This phenomenon indicated that the roughness of the joint and the initial normal stress remained unchanged, and the strength of the shear deformation resistance of the specimen was mainly affected by the initial shear stress. The larger the value of initial shear stress was, the larger the normal stress required by the joint to resist failure was, that is, the smaller the unloading amount was. The unloading amount reflected the degree of failure of joints. The larger the unloading amount was, the more difficult the failure of joints was.

**Figure 14:**
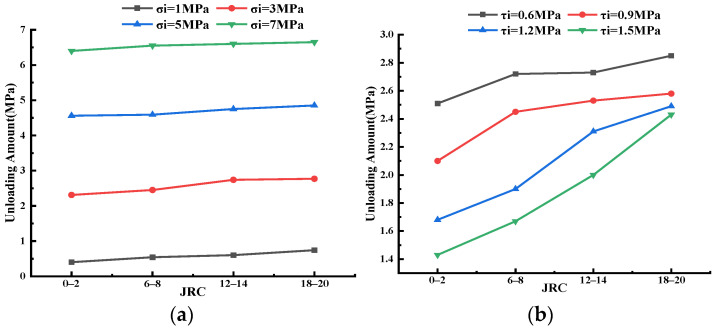
Unloading amount and roughness. (**a**) Group A. (**b**) Group B.

Figure 14 shows the relationship between unloading amount and roughness. Figure 14a is Group A (in Table 2). The unloading amount of specimens varied little and presented an overall upward trend under different roughness of joints. However, with the increase in σ_i_, the increase in unloading decreased gradually. When σ_i_ was 1 MPa and JRC was 0–2, the unloading amount was 0.4 MPa, and when JRC was 18–20, the unloading amount was 0.74 MPa, with an increase of 85.0%. When σ_i_ was 3 MPa, 5 MPa, and 7 MPa, the increase decreased to 19.9%, 6.4%, and 3.9%, respectively. Figure 14b is Group B. As the roughness increased, the unloading amount gradually increased and the change was obvious. When τ_i_ was 0.6 MPa and JRC was 0–2, the unloading amount was 2.51 MPa, and when JRC was 18–20, the unloading amount was 2.85 MPa and the increase was 13.5%. The increase in τ_i_ was 22.9%, 48.2% and 69.9% at 3, 5 and 7 MPa, respectively. According to Group A and Group B, the larger JRC was, the more difficult it was for the specimen to fail under normal unloading conditions. In Group A, τ_i_ was small (0.7 MPa), which made the unloading amount insensitive to roughness. In Group B, τ_i_ was 0.6, 0.9, 1.2 and 1.5 MPa, respectively, which made the unloading amount of the specimen sensitive to the change in roughness in the shear process.

### 3.3. Laws of Acoustic Emission

**Figure 15:**
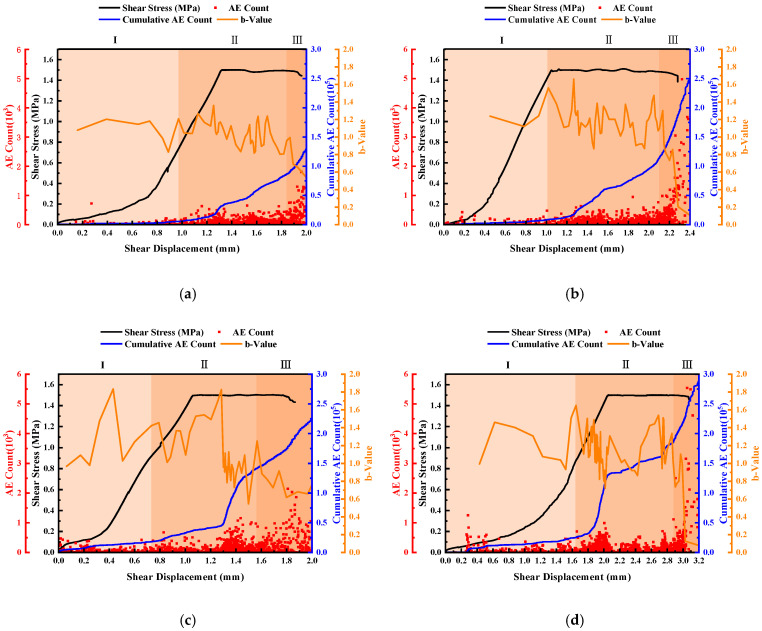
Acoustic emission rules of joints with different roughness. (**a**) JRC = 0–2. (**b**) JRC = 6–8. (**c**) JRC = 12–14. (**d**) JRC = 18–20.

The phenomenon of material rapidly releasing energy and generating transient elastic waves due to local regional stress concentration is called acoustic emission [48]. In this section, the relationship between AE event rate, cumulative AE event number, *B* value and joint roughness was analyzed during the whole process from the initiation of shear stress to the final joint failure (i.e., the sudden decrease of shear stress). Figure 15 shows the acoustic emission laws of different roughness of joints when σ_i_ was 3 MPa and τ_i_ was 1.5 MPa. In general, the unloading shear acoustic emission process can be divided into a quiet period, a stable growth period and a failure period, corresponding to stages I, II and III in Figure 15, respectively.

Firstly, the AE event rate and cumulative AE event number were analyzed. The variation law of the AE event rate and cumulative AE event number with shear displacement was consistent under different roughness. In stage I, the AE event rate was low, the continuity was poor, the cumulative AE events were few, and the curve was flat and concave. According to the shear stress–displacement curve, at this time, the joint was in the compaction and elastic deformation stage, and its internal microcracks initiated and expanded less [49,50]. In stage II, the AE event rate increased with good continuity, and the slope of the cumulative AE events–shear displacement curve increased, indicating that the microcracks in the specimen generated uniformly, expanded more and had strong continuity. In stage III, the AE event rate increased suddenly and reached a peak value. The duration was shorter, and the continuity was weaker than that in stage II. The cumulative AE events–shear displacement curve was more steep, and the curve rose rapidly to the joint to cause through failure. This phenomenon showed that when the normal stress was unloaded to the failure normal stress, the joint suddenly lost stability. With the increase in roughness, the cumulative number of AE events also increased when the joint finally broke through. When JRC was 0–2, the cumulative number of AE events during joint failure was 110,513, and when JRC was 18–20, the cumulative number of AE events reached 249,926. This is because the coarser the joint, the stronger the cementation between particles, the greater the shear strength of the joint, and the greater the energy required for the penetration failure of the joint [51]. This is similar to the rule of cumulative events in direct shear with different roughness [52].

The *b*-value is closely related to the initiation and development of cracks in rocks. A large *b*-value indicates that a large number of small fracture events occur in the rock, while a small *b*-value indicates that large cracks occur in the rock; i.e., the more serious the damage. An increase in *b*-value indicates the enhancement of acoustic emission activity in the process of rock loading, which is mainly characterized by small-scale micro-fracture. A decrease in *b*-value indicates that large-scale cracks occur or crack propagation speed increases sharply. When the *b*-value is constant, the distribution of large- and small-scale micro-fracture phenomena in the rock is balanced [53]. The calculation formula of the *b*-value is

(6) lgN=a−b(A/20)

where *b* is the physical quantity characterizing the activity level of acoustic emission, *A* is the amplitude of acoustic emission, *N* is the statistical cumulative frequency of acoustic emission under the magnitude interval, and *a* is an empirical constant.

The *b*-value fluctuated up and down with the increase in shear displacement in Figure 15. In stage I, the *b*-value showed an upward trend on the whole; the *b*-value curve was sparse and the amount value was large, and the fluctuation range of the amount value was 0.97–1.65. This is because at the initial stage of shear stress loading, the tiny convex body on the joint, which was dominated by small-scale micro-fractures, was first cut off. In stage II, the *b*-value fluctuated sharply in a certain range and decreased on the whole, and the *b*-value curve was dense. This is because with the increase in shear stress and in the normal unloading process, the small convex body on the joint was constantly cut off, the microcracks were continuously connected into large cracks, the small and large events appeared alternately and the proportion of large events increased. At this time, the small convex body on the joint was basically cut off. In stage III, the *b*-value showed a decreasing trend, and at the last stage when the failure was about to occur, the *b*-value dropped sharply and the *b*-value curve was sparse. In this stage, with the decrease in normal stress, the joint suddenly failed at a certain moment, and the large protrusions on the joint were cut off. This stage was dominated by large-scale fracture events. It can be observed that, with the increase in the roughness of the joint, the more severe the fluctuation of the *b*-value in stage II was and the more dense the curve was. This phenomenon indicated that the rougher the joint was, the more frequent the alternation of magnitude events in the shear process of the joint was.

It is worth noting that in some shear tests carried out under the conditions of constant normal stress, researchers divided the whole AE process into three stages: the quiet phase, the stable growth phase and the rapid growth phase [54,55,56]. Comparative analysis showed that AE events in the first stage of normal unloading test are fewer, which was similar to the phenomenon of AE in the first stage of direct shear testing under constant normal stress [54]. This indicated that at the beginning of the test, the internal damage of rock mass was relatively small due to the small value of the shear stress. In the second stage of AE, the *b*-value fluctuated frequently. This phenomenon was more obvious in the direct shear test under normal unloading conditions [55]. When the shear stress reached the target value, with the decrease in the normal stress, the joint was more prone to slide in the shear direction, resulting in the alternating of large and small fracture events in the rock mass. Under the conditions of constant normal stress, the internal damage process of rock mass was relatively stable because the shear stress loading rate remained unchanged. The *b*-value fluctuated up and down, but the frequency was relatively slow. In the third stage of AE, the maximum AE event rate value appeared at the moment when the shear stress reached a peak [56]. Under the conditions of normal unloading, the maximum AE event rate value appeared at the moment when the normal stress was unloaded to the failure normal stress. The above phenomena indicated that under normal unloading conditions, the rock mass is unstable and the failure is more sudden.

## 4. Energy Evolution Law of Normal Unloading

### 4.1. Energy Analysis of Normal Unloading Test

Conservation of energy is one of the basic laws of natural science, and is also applicable to geotechnical engineering. In the process of rock compression, tension, shear and impact, there are changes in energy. In the unloading shear process of joints, the energy conversion process is divided into the following three steps: (1) The normal stress loading stage. At this stage, with an increase in normal stress, the joint kept compacting and the normal displacement increased. Ignoring the lateral expansion of the specimen, the normal stress did positive work to the joint. At this point, no external force was applied in the shear direction, and the work done by the shear force on the joint was 0 J. (2) The shear stress loading stage. At this stage, the normal stress remained constant and the shear stress increased. As the shear stress increased, the shear displacement began to increase, and the shear stress did positive work on the joint. At this point, due to dilatancy of the specimen, the normal stress exerted negative work on the joint, and the total work of the normal stress on the joint decreased. (3) The normal stress unloading stage. At this stage, the shear stress remained constant and the normal stress began to decrease until the specimen failed. As the normal stress decreased, the normal displacement continued to decreased, and the normal stress continued to do negative work on the joint. At this point, the shear displacement increased due to the presence of shear stress, which did positive work to the joint.

**Figure 16:**
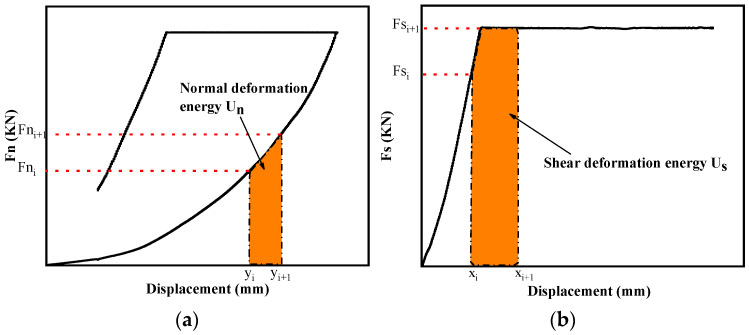
Calculation of energy by differential method. (**a**) Normal deformation energy U_n_. (**b**) Shear deformation energy U_s_.

The normal deformation energy U_n_ was calculated according to the normal force–displacement curve (Figure 16a), and the shear deformation energy U_s_ was calculated according to the shear force–displacement curve (Figure 16b). The total energy U_0_ was equal to U_n_ plus U_s_.

The calculation formula is:

(7) $$U=\int_{0}^{x_{i}}Fdx$$

For the integral of Equation (7), the sum of small trapezoidal area can be adopted according to the definition of definite integral. Since the time interval T_0_ of data collection is 0.05 s, the height of the tiny trapezoid is the displacement difference of each 0.05 s, then

(8) $$\begin{array}{l} U_{n}=\sum_{i=0}^{n}\frac{1}{2}(Fn_{i}+Fn_{i+1})(y_{i+1}-y_{i})\\ U_{s}=\sum_{i=0}^{n}\frac{1}{2}(Fs_{i}+Fs_{i+1})(x_{i+1}-x_{i}) \end{array}\}$$

where *Fn_i_* and *Fs_i_* are the normal force and shear force borne by the rock mass at time *i*, respectively, and *y_i_* and *x_i_* are the normal displacement and shear displacement of the rock mass at time *i*, respectively. The same applies at time *i* + 1.

According to the above analysis, U_0_ is equal to U_s_ plus U_n_, which can be obtained by combining Equation (8):

(9) $$U_{0}=\sum_{i=0}^{n}\frac{1}{2}[(Fn_{i}+Fn_{i+1})(y_{i+1}-y_{i})+(Fs_{i}+Fs_{i+1})(x_{i+1}-x_{i})]$$

### 4.2. Energy Evolution Curve of Unloading Test

As the initial stress of each specimen was different during unloading, the energy states of the joint at the unloading point were different. The energy values at unloading under different initial stress conditions are shown in Table 4. Groups I and II in Table 4 are representative specimens with different σ_i_ and τ_i_, respectively. According to the specimens in Group I, when the τ_i_ and the roughness of the joint were the same, the total energy of the specimen at the unloading point would increase gradually with the increase in σ_i_. This was because in the normal loading phase, the larger the σ_i_, the closer the joint would be compressed, and the more positive work would be done by the normal force. In the process of applying shear stress, although there would be dilatancy of joints, with the increase in σ_i_, the dilatancy became less obvious, i.e., in the tangential loading stage, the negative work done by normal stress would be less. Therefore, the greater σ_i_ are, the greater the total energy before unloading. According to specimens in Group II, when σ_i_ and roughness were the same, the total energy of the specimen before unloading increased with the increase in τ_i,_ but the increase was relatively small. This is because in the normal stress loading stage, as σ_i_ applied by each group was the same, the positive work done by the normal stress on the joint was basically the same. In the tangential stress loading process, the specimens were under the same normal stress at this time. With the increase of τ_i_, the specimen would eventually produce more shear displacement, and the more positive work the shear stress did. Although there will be dilatancy on the joint, the negative work done by the normal stress at this stage was smaller than the positive work done by the shear stress. Therefore, the larger τ_i_ was, the greater the total energy before unloading would be.

**Figure 17:**
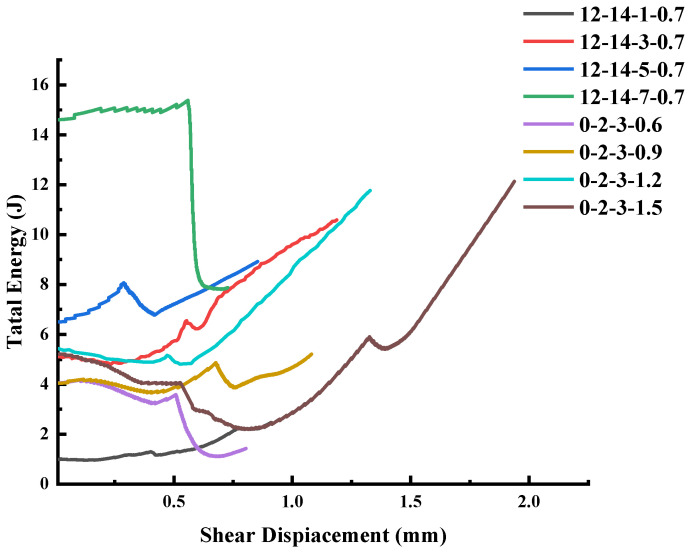
Total energy.

**Figure 18:**
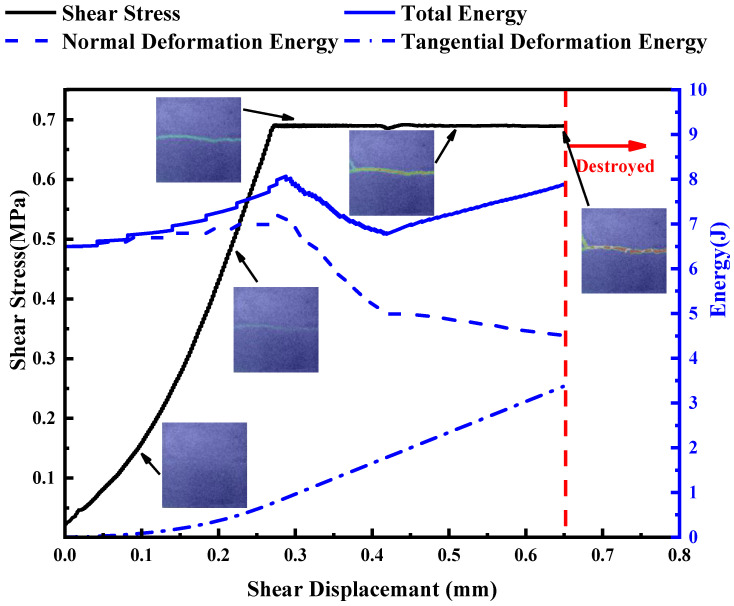
Curves of energy, shear stress and shear displacement.

Figure 17 shows the total deformation energy–shear displacement curve. In this paper, a specimen with σ_i_ of 5 MPa, τ_i_ of 0.7 MPa and JRC of 12–14 was taken as an example to illustrate the energy characteristics of the joints’ instability failure under normal unloading conditions, as shown in Figure 18.

It can be seen from Figure 18 that in the first stage, when the normal stress reached σ_i_ (5 MPa), both U_0_ and U_n_ were 6.49 J, and the shear displacement basically did not change at this time. In the second stage, the normal stress remained unchanged and shear stress was applied. U_s_ increased gradually with the increase in shear displacement, and the growth rate also increased gradually. This is because in the initial shear stage, the shear stiffness of the joint was small, and the shear stress increment was small under the same shear displacement increment, so U_s_ increased slowly. With the increase in shear stress, the shear stiffness of joints increased gradually when the joint was fully in contact. The greater the shear stress required for the same shear displacement, the faster the U_s_ increased. After the shear stiffness was basically stable, the U_s_ increment under the same shear displacement increment was also basically stable, and both of then showed a linear relationship. Due to the large roughness of the joint, when the shear stress was applied, U_n_ would increase by 0.69 J, which was about 10.6% of U_n_ before the shear shrinkage. During this stage, U_0_ increased continuously, eventually reaching 8.07 J. In the third stage, the normal stress began to decrease, while the shear stress remained unchanged. After the normal unloading began, U_n_ decreased, U_s_ continued to increase, and U_0_ decreased first and then increased. U_0_ increased from the minimum point, and soon after, the joint became unstable and failed. Therefore, it can be inferred that in the unloading process, when U_0_ changes from decreasing to increasing, it indicates that unloading instability failure of joints will occur, and this law is conducive to predicting engineering rock mass disasters [57,58]. U_0_ changed from decreasing to increasing, which indicated that the positive work done by shear stress was greater than the negative work done by normal stress. At this time, the rock mass had a large displacement in the shear direction, and the normal stress was not enough to prevent the sliding of the rock mass, indicating that the rock mass is about to fail.

In order to have a deeper understanding of the law of energy evolution, DIC was combined with energy evolution in this section. It can be seen from Figure 18 that when shear stress was applied at the beginning, the principal strain distribution on the surface of rock mass was uniform. The surface of the rock mass passed through the uneven zone when the shear stress reached the initial shear stress. The color of the uneven zone became darker when the rock mass was near to instability (i.e., U_0_ changed from decreasing to increasing). When the rock mass was unstable, a high strain zone passed through the rock mass surface.

Energy conversion runs through the whole process of rock deformation and failure, so many scholars have recorded rock failure behavior from the perspective of energy analysis. These studies focus on the variation law between dissipated energy, elastic strain energy and total energy [31,59,60]. Loading methods and rock properties all affect the variation of energy. Some scholars have also proposed failure criteria based on energy conversion. For example, Xiao, Yu [23] proposed a fatigue life prediction model based on generalized strain energy density. Zhu, Huang [61] characterized rock damage characteristics through the dissipated energy of rock mass in the shear process. According to the evolution of total energy, Zhai, Guo [47] predicted the instability of rock with perforated fissure under compression–shear conditions, which produced results similar to those in this paper. This also shows that it is feasible to predict rock mass instability by total energy under certain conditions. Of course, the prediction of rock mass instability based on total energy in this paper has limitations. For example, it may no longer be applicable under complex stress conditions. We will improve this method in future studies. In the following studies, we will further consider the evolution law of elastic strain energy and dissipated energy [61,62,63], and study the influence of factors such as scale effect [64] and coupling effect [65] on the shear mechanical behavior and energy evolution law of rock mass under normal unloading conditions, so as to further enrich the mechanics of unloaded rock mass.

## 5. Conclusions

(1) The shear failure of specimens under normal unloading σ_f_ was greater than the corresponding value of conventional direct shear test σ_p_. The unloading mode of keeping the shear stress unchanged and reducing the normal stress weakened the shear capacity of the joint. The unloading amount increased with the increase in initial normal stress and roughness, and decreased with the increase in initial shear stress.
(2) The larger the JRC, the stronger the climbing effect of the joint, but the increase in the initial stress will enhance the tooth cutting effect. The higher the initial normal stress and JRC, the longer the time of the high strain zone on the rock surface, and the higher the initial shear stress, the earlier the time of the high strain zone on the rock surface.
(3) The variation law of AE events and the *b*-value at each stage was different from that under constant normal stress, which indicated that it was necessary to carry out shear testing under normal unloading conditions. Additionally, the greater the roughness of the joint was, the larger the cumulative AE events were, and the denser the *b*-value curve was in stage II.
(4) A new method for predicting the instability of joints under normal unloading conditions was proposed based on the evolution law of U_n_, U_s_ and U_0_. In the unloading process, U_0_ changed from decreasing to increasing, indicating that the joint would be unstable due to unloading.

## Figures and Tables

**Table 1:** Mechanical parameters of joint.

Roughness	Cohesion/MPa	Internal Friction Angle/°
0–2	0.214	37.5
6–8	0.328	41.0
12–14	0.592	42.8
18–20	0.844	42.6

**Table 2:** Test scheme.

Group	Initial Stress/MPa		Roughness
	σ_i_	τ_i_	
A	1	0.7	0–2 6–8 12–14 18–20
	3	0.7	
	5	0.7	
	7	0.7	
B	3	0.6	
	3	0.9	
	3	1.2	
	3	1.5	

**Table 3:** Specimen parameters.

Group	Number	Roughness	σ_i_/MPa	τ_i_/MPa
Ⅰ	S1	0–2	3	1.2
	S2	6–8	3	1.2
	S3	12–14	3	1.2
	S4	18–20	3	1.2
Ⅱ	S5	12–14	1	0.7
	S6	12–14	3	0.7
	S7	12–14	5	0.7
	S8	12–14	7	0.7
Ⅲ	S9	12–14	3	0.6
	S10	12–14	3	0.9
	S11	12–14	3	1.2
	S12	12–14	3	1.5

**Table 4:** Total energy of unloading point under different initial stress conditions.

Group	σ_i_/MPa	τ_i_/MPa	JRC	Total Energy of Unloading Point/J
Ⅰ	1	0.7	12–14	2.23
	3	0.7	12–14	6.54
	5	0.7	12–14	8.07
	7	0.7	12–14	15.37
Ⅱ	3	0.6	0–2	3.53
	3	0.9	0–2	4.82
	3	1.2	0–2	5.16
	3	1.5	0–2	5.84

## Data Availability

The data used to support the findings of this study are available from the corresponding author upon request.
